# Sex‐biased breeding dispersal is predicted by social environment in birds

**DOI:** 10.1002/ece3.4095

**Published:** 2018-06-21

**Authors:** Zsolt Végvári, Gergely Katona, Balázs Vági, Robert P. Freckleton, Jean‐Michel Gaillard, Tamás Székely, András Liker

**Affiliations:** ^1^ Department of Conservation Zoology Debrecen University Debrecen Hungary; ^2^ Hortobágy National Park Directorate Debrecen Hungary; ^3^ Department of Evolutionary Zoology University of Debrecen Debrecen Hungary; ^4^ Department of Animal and Plant Sciences University of Sheffield Sheffield UK; ^5^ Unité Mixte de Recherche 5558 “Biométrie et Biologie Evolutive” Université de Lyon Villeurbanne Cedex France; ^6^ Department of Biology and Biochemistry Milner Centre for Evolution University of Bath Bath UK; ^7^ Wissenschaftskolleg zu Berlin Berlin Germany; ^8^ MTA‐PE Evolutionary Ecology Research Group University of Pannonia Veszprém Hungary; ^9^ Department of Limnology University of Pannonia Veszprém Hungary

**Keywords:** adult sex ratio, comparative analysis, mortality, natal dispersal, sex role, sexual selection

## Abstract

Sex‐biased dispersal is common in vertebrates, although the ecological and evolutionary causes of sex differences in dispersal are debated. Here, we investigate sex differences in both natal and breeding dispersal distances using a large dataset on birds including 86 species from 41 families. Using phylogenetic comparative analyses, we investigate whether sex‐biased natal and breeding dispersal are associated with sexual selection, parental sex roles, adult sex ratio (ASR), or adult mortality. We show that neither the intensity of sexual selection, nor the extent of sex bias in parental care was associated with sex‐biased natal or breeding dispersal. However, breeding dispersal was related to the social environment since male‐biased ASRs were associated with female‐biased breeding dispersal. Male‐biased ASRs were associated with female‐biased breeding dispersal. Sex bias in adult mortality was not consistently related to sex‐biased breeding dispersal. These results may indicate that the rare sex has a stronger tendency to disperse in order to find new mating opportunities. Alternatively, higher mortality of the more dispersive sex could account for biased ASRs, although our results do not give a strong support to this explanation. Whichever is the case, our findings improve our understanding of the causes and consequences of sex‐biased dispersal. Since the direction of causality is not yet known, we call for future studies to identify the causal relationships linking mortality, dispersal, and ASR.

## 1 INTRODUCTION

Dispersal is an essential part of life history in many organisms that has knock‐on consequences for demography, ecology, and behavior. By moving from one area to another, dispersal also influences meta‐population dynamics and gene flow and thus has implications for diversification rates and responses of animals to environmental changes by shifting their geographic ranges (Bowler & Benton, 2005; Clobert, 2012; Clobert, Danchin, Dhondt, & Nichols, 2001; Morales et al., 2010).

The costs and the benefits of dispersal may differ between the sexes due to differences in ecology, behavior, and physiology between males and females. Therefore, the frequency and extent of dispersal movements tend to be sex‐biased (Clobert et al., 2001). Indeed, sex‐biased dispersal has been documented for several taxa including invertebrates, reptiles, birds, and mammals (Clobert et al., 2001; Greenwood, 1980; Trochet et al., 2016), although its underlying ecological and evolutionary causes and consequences are still poorly known. For instance, in birds, females tend to disperse farther than males, whereas in mammals, the reverse appears to be the case (Greenwood, 1980; Clarke, Sæther, & Røskaft, 1997; Mabry, Shelley, Davis, Blumstein, & Van Vuren, 2013.)

In understanding the ecology and evolution of dispersal, it is important to distinguish between two distinct types of dispersal. First, natal dispersal occurs when individuals settle their own home range before their first breeding (i.e., when they move from their natal area to their future breeding area). Second, breeding dispersal occurs when individuals move from their former breeding area to a new breeding site. Since natal and breeding dispersal are likely driven by different biological mechanisms (Clobert, 2012), sex biases in these dispersal types may also be related to different ecological and/or evolutionary factors.

Sex‐biased natal and breeding dispersal may develop for several reasons. First, sex‐biased dispersal can evolve in response to sexual selection because sex differences in reproductive behavior might lead to sex differences of spatial distribution (Greenwood, 1980; Pusey, 1987). Thus, the more polygamous sex is expected to disperse farther because members of that sex may experience strong intrasexual competition and should move long distances to find new mating partners (Emlen & Oring, 1977; Jackson et al., 2017; Kempenaers & Valcu, 2017; Trochet et al., 2016). However, comparative analyses of the relationship between the magnitude of sex differences in dispersal and mating systems performed so far failed to support this hypothesis (Clarke et al., 1997; Mabry et al., 2013; Trochet et al., 2016). In addition, since the intensity of sexual selection is usually associated positively with sexual size dimorphism (SSD), plumage dimorphism and/or different extent of male vs. female parental care (e.g., Andersson 1994), the magnitude of sex differences in dispersal should also be positively related to SSD, plumage dimorphism, and the amount of male care relative to female care. Although sex‐biased dispersal can be related to several of the above components of sexual selection, previous studies have mainly focused on mating system and SSD (e.g., Mabry et al., 2013).

Second, the social environment may also induce sex‐biased dispersal. In species with biased adult sex ratio (ASR), members of the more common sex should seek out new breeding sites to avoid local resource or mate competition. Furthermore, dispersal can be costly in terms of survival. For instance, individuals of the more dispersive sex have been reported to suffer from higher predation rates than those of the less dispersive sex (e.g., Steifetten & Dale, 2012). Such mortality effects of sex‐specific dispersal may in turn influence ASR. Although previous studies suggest that biased ASRs are associated with sex‐biased adult mortality (Székely, Liker, Freckleton, Fichtel, & Kappeler, 2014a), the relationship between ASR and sex‐biased dispersal is poorly known (but see Pipoly et al. (2015) for preliminary analyses). The complexity of this relationship was illustrated by a study of butterflies showing that inter‐ and intrasexual aggression induced higher dispersal rates of both sexes from populations where the proportion of males was experimentally increased (Trochet et al., 2013)

Third, sex‐biased natal dispersal is often thought as a mechanism to avoid inbreeding depression. If one gender disperses further than the other, the chances of close relatives breeding together and suffering the costs of inbreeding are reduced (Auld & de Casas, 2013; Clutton‐Brock, 2016). For example, higher inbreeding results in an increase in homozygosity, which is associated with fitness loss and with inbreeding depression in normally outbred populations (Pusey, 1987). However, recent investigations only reported weak effects of inbreeding avoidance on the direction of sex‐biased dispersal (Guillaume & Perrin, 2009; Trochet et al., 2016).

We investigated here the first two of the above hypotheses, namely the potential influence of the intensity of sexual selection and of the social environment on the magnitude of sex‐biased natal and breeding dispersal (defined here as the difference between male and female dispersal distances), across 86 bird species from 41 avian families. Although some comparative studies of avian dispersal have been previously performed, they were either qualitative (Clarke et al., 1997; Greenwood, 1980) or did not distinguish between natal and breeding dispersal (Mabry et al., 2013; Trochet et al., 2016). We aim to fill this knowledge gap by specifically testing whether the magnitude and the direction of sex‐biased natal and breeding dispersal in birds are positively associated with the intensity of sexual selection (using several metrics including social mating system, frequency of extra‐pair paternity (EPP), SSD, and plumage coloration) and with the amount of sex differences in parental care. In addition, we test whether sex differences in dispersal are related to the social environment (using ASR as a proxy). This latter relationship may involve either the influence of ASR on dispersal (i.e., individuals of the more common sex should seek out new breeding sites to avoid intraspecific competition) or the existence of dispersal costs in terms of mortality (i.e., the farther dispersing sex should suffer from higher mortality, which should lead to biased ASR).

## 2 MATERIALS AND METHODS

### 2.1 Sex‐specific natal and breeding dispersal distances

We collected sex‐specific dispersal data (both from census and capture–recapture studies) by searching primary publications in peer‐reviewed journals and books and also by tracking back references cited by previous reviews and phylogenetic analyses (Mabry et al., 2013; Trochet et al., 2016; for further details, see Appendices S1–S3). Following Clobert (2012), we defined natal dispersal distance as the average movement of individuals from their birth site to the site of reproduction. Breeding dispersal distance was defined as the average movement of individuals between successive sites of reproduction. Both dispersal distances were measured in kilometers.

We extracted data on mean dispersal distances separately for each sex and defined dispersal sex bias as log(male dispersal distance) – log(female dispersal distance). We calculated these sex biases separately for natal and breeding dispersal, and we termed these variables “natal dispersal bias” and “breeding dispersal bias,” respectively. Male and female dispersal data were only used if they were estimated from the same population. If sex‐specific estimates were available for several populations within a given species (see Appendix S1), we used the unweighted means of all populations, since for other variables of interest we only had species‐level information. In total, we obtained data on natal dispersal for 64 species from 32 avian families and on breeding dispersal for 41 species from 28 families. We did not retain qualitative statements such as “males disperse further than females” in our analysis (Appendix S3).

We looked for the consistency between our dataset and those collected by Mabry et al. (2013) and Trochet et al. (2016) who reported information on 56 and 46 sex‐specific dispersal distances in birds, respectively. Note that neither Mabry et al. (2013) nor Trochet et al. (2016) distinguished between natal and breeding dispersal. Nonetheless, the three datasets are correlated, as shown by Pearson's correlation coefficients between our log‐transformed metrics of dispersal bias and the log‐transformed metrics of dispersal bias used by Mabry et al. (2013) and Trochet et al. (2016) (*r* = .507 and .999, *p* < .001 and *p* < .0001, *N* = 21 and 35 species for natal and breeding dispersal, respectively).

### 2.2 Predictors of sex‐biased dispersal

We defined different metrics of sexual selection including:

1. The social mating system bias, measured as male minus female scores for the degree of social polygamy, where we defined the scores for each sex on a scale ranging between 0 and 4, where zero corresponds to no (or very rare) social polygamy (<0.1% of individuals), 1 to rare polygamy (0.1–1%), 2 to uncommon polygamy (1–5%), 3 to moderate polygamy (5–20%), and 4 to common polygamy (>20%; including males in lekking species to express the high variance in male mating success in these species; Liker, Freckleton, Remeš, & Székely, 2015). Thus, a positive value in mating system bias means higher frequency of male social polygamy relative to the frequency of female polygamy,
2. The proportion of broods containing extra‐pair offspring,
3. Relative testes mass, calculated as log(testis mass) – 0.67*log(male mass), where 0.67 is the allometric exponent estimated by Møller (1991) from a large range of bird species,
4. SSD, expressed as log(male mass) – log(female mass) when assuming an isometric relationship between male and female mass,
5. The degree of dichromatism, calculated using the scoring system based on Owens and Hartley (1998), using the following rules. Each species was split into five main body regions (head; nape, back, and rump; throat, chest, and belly; tail; and wings), shortly referred to as head, back, belly, tail, and wings, respectively. The score used ranged between −2 and 2, where −2 means that females are substantially brighter and/or more patterned than males; −1 means that females are brighter and/or more patterned than males; 0 means that there is no difference in the body region or the difference is too tiny to assess that one sex is brighter than the other; 1 means that males are brighter and/or more patterned than females; and 2 means that males are substantially brighter and/or more patterned than females. The mean of these scores was recorded, as well as an overall score, which was the sum of all the scores. A single observer scored the ornamentation of all species using the illustrations from del Hoyo, Elliott, Sargatal, Christie, and de Juana (2016). The repeatability of the scoring was estimated by the same observer blind to species ID. The two scorings yielded high consistency of measurements (minimal and maximal values of Spearman rank correlation: ρ_min_ = 0.794, ρ_max_ = 1.000, *p* < .0001 for all cases).

To estimate sex bias in parental care, we scored the relative participation of the sexes in each of six care components: nest building, incubation, nest guarding, brooding, chick feeding, and chick guarding prior to the fledging of the chicks. We used a 5‐point scale, with positive scores meaning more male than female care and negative scores meaning more female than male care (−1: no male care, −0.5: 1–33% male care, 0: 34–66% male care [i.e., equal or similar care by the sexes], 0.5: 67–99% male care, 1: 100% male care; Székely et al., 2014a; Liker et al., 2015). These scores were based on quantitative data when available (e.g., percentage of incubation by males), or on qualitative descriptions of care in the data source when quantitative data were not available. As we did not find data for all care components for all species, the actual number of care components on which these mean scores were based differed among species. Note that mean scores calculated from a given set of care components correlated strongly with mean values of other sets of care components (see Liker et al., 2015 for details).

Adult sex ratio was calculated as the arcsine‐transformed proportion of males in the adult populations. Since ASR estimation is often error‐prone (Székely, Weissing, & Komdeur, 2014b), we studied the potential confounding effect of the method of ASR estimation and for this purpose ASR method was categorized as a two‐level factor (census vs. capture). As a potential driver of the relationships between dispersal sex bias and ASR, we also collected data on annual adult mortality sex bias that was calculated as log(adult male mortality) – log(adult female mortality) (Székely et al., 2014b). All log‐transformations applied 10‐based logarithmic functions.

### 2.3 Phylogenetic analyses

To assess whether natal dispersal and/or breeding dispersal differed between sexes, we conducted phylogenetic paired *t* tests. We computed the so‐called phylogenetic mean for the difference between two values (i.e., two sexes) of each species and tested whether the mean difference was statistically different from zero (Lindenfors, Revell, & Nunn, 2010). We used the implementation of this test provided in R package “phytools” (Revell, 2012).

We used phylogenetic least‐squares (PGLS) analysis to investigate relationships between sex‐specific natal and breeding dispersal as well as predictors related to sexual selection and social environment as specified by the hypotheses (Freckleton, Harvey, & Pagel, 2002; Martins & Hansen, 1997; Pagel, 1999). This approach allows controlling for the nonindependence among species by incorporating a variance–covariance matrix that represents their phylogenetic relationships. In all analyses, we set the phylogenetic signal (λ) to the maximum‐likelihood value (Freckleton et al., 2002). To test phylogenetic signal in the sign of sex‐biased dispersal, we retrieved the values of λ from PGLS models and performed *D*‐statistics, a further measure of the strength of phylogenetic signal, presented in Appendix S5. *D* provides a measure of the phylogenetic signal in a binary trait, calculated as the sum of changes in estimated nodal values of that trait along edges in a phylogeny (Fritz and Purvis, 2010). Specifically, *D*‐test compares the observed *D*‐value for a binary trait on a tree to the value of *D* found using an equal number of simulations considering each of two models: (1) phylogenetic randomness, where trait values are randomly permuted among the tips of the phylogeny (*D* = 1), and (2) Brownian threshold model, where a continuous trait evolved along the phylogeny following Brownian process and then converted to a binary trait using a threshold providing the relative prevalence of the observed trait (*D* = 0). To test whether *D* differed from phylogenetic randomness, we computed *p*‐values for *D* = 1 (P1). As a result, we detected only low levels of phylogenetic signal in the sign of the sex‐specific dispersal, shown both by low values of λ and by P1 > .059 for all cases (Appendix S4).

We tested pairwise relationships between both natal and breeding dispersal biases (dependent variables) and each dispersal predictor if data were available for at least 10 species. In all these PGLS analyses, we ran each model with 100 random phylogenetic trees retrieved from BirdTree.org (http://www.birdtree.org; Jetz, Thomas, Joy, Hartmann, & Mooers, 2012). These composite time‐calibrated trees were pruned to keep only the species used in the analyses (Paradis, Claude, & Strimmer, 2004). We computed mean ± *SE* for slopes, where *SE*‐s were computed as the square root of the total variance, defined as the sum of the average parameter variance ($V_{1}^{2}$ = 1/N*Σ($SE_{i}^{2}$)) and phylogenetic variance ($V_{2}^{2}$ = *SE*(*b*)^2^), calculated across the 100 runs. We retrieved *p*‐values from the results of the 100 runs and reported *SE* of the 100 *p*‐values.

Some of the species in our dataset came from hunted populations, which may influence the dispersal behavior of one or both sexes. To test the sensitivity of our results to this potential effect, we repeated all analyses with the exclusion of the hunted species and reported the multitree‐averaged parameter estimates and adjusted R^2^‐s to assess the consistency of effect sizes (Appendix S5).

All PGLS analyses were run with R 3.1.0 (R Core Development Team 2014), using the “caper” package (Orme, 2013). Sample sizes differed between analyses because for many species data were available only for a subset of the variables. The full dataset including the references will be made available in an open‐access data depository once the manuscript is accepted for publication (http://www.openbiomaps.org).

## 3 RESULTS

### 3.1 Sex difference in dispersal

**Figure 1:**
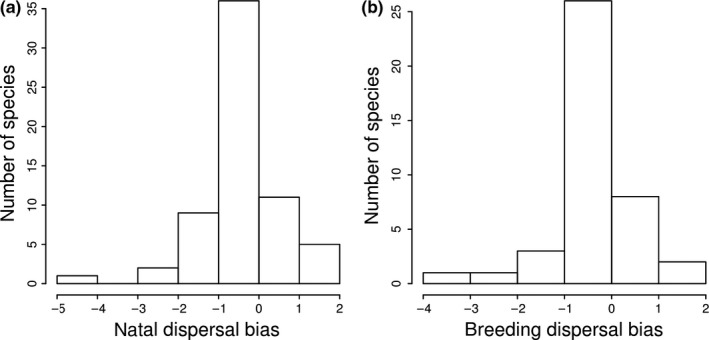
Distribution of (a) natal and (b) breeding dispersal bias in birds, calculated as difference between log‐transformed male and female dispersal distances (in km). *N* = 64 and 41 species in (a) and (b), respectively

There was a statistically significant sex bias in both natal and breeding dispersal: Females dispersed further away than did males (phylogenetic paired *t* tests, natal dispersal: *p* = .016 ± .0001, *N* = 64 species; breeding dispersal: *p* = .010 ± .0001, *N* = 41 species, Figure 1a,b). Interestingly, the sex biases in natal and breeding dispersal were not related to each other (PGLS, *b* = 0.066 ± 0.076, *p* = .895 ± .0001, *N* = 19 species; Appendix S6).

### 3.2 Sex‐biased dispersal and the intensity of sexual selection

Sex bias in either natal or breeding dispersal was not related to any metric measuring the intensity of sexual selection (Table 1). In addition, sex‐specific parental care was unrelated to either natal or breeding dispersal bias (Table 1).

**Table 1:** Sex‐biased dispersal (response variable) in birds in relation to the intensity of sexual selection and to the social environment using phylogenetic least‐squares models. Table shows parameter estimates calculated using 100 phylogenies (see Methods for further explanation). N refers to the number of species

Predictors	Natal dispersal bias		Breeding dispersal bias	
	*b* ± *SE*	*p* ± *SE* N	*b* ± *SE*	*p* ± *SE* N
Sexual selection				
Social mating system	0.026 ± 0.074	.476 ± 0.005, 58	−0.013 ± 0.114	.764 ± 0.013, 28
Testis size	−0.076 ± 0.191	.694 ± 0.0001, 36	−0.297 ± 0.791	.0948 ± 0.0001, 29
Extra‐pair broods	−0.443 ± 0.477	.361 ± 0.004, 36	0.387 ± 0.397	.5502 ± 0.01, 27
Parental care	0.041 ± 0.111	.798 ± 0.0001, 56	−0.151 ± 0.169	.256 ± 0.0001, 30
Sexual size dimorphism	0.401 ± 1.001	.693 ± 0.0001, 55	0.234 ± 1.009	.551 ± 0.0001, 38
Sexual dichromatism	0.033 ± 0.087	.710 ± 0.0001, 25	0.045 ± 0.0932	.599 ± 0.0001, 22
Social environment				
Adult sex ratio	−31.740 ± 25.234	.223 ± 0.0001, 24	−68.376 ± 28.713	.0411 ± 0.0001, 14
Mortality bias	Not tested	Not tested	2.067 ± 0.031	.0593 ± 0.002, *N* = 25

### 3.3 Sex‐biased dispersal and social environment

**Figure 2:**
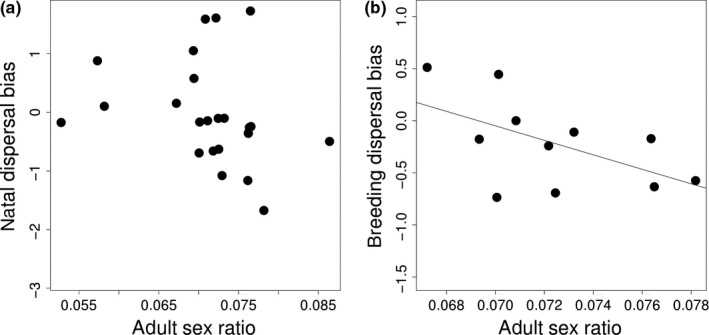
Adult sex ratio in relation to (a) natal dispersal bias (*N* = 24 species) and (b) breeding dispersal bias (*N* = 14 species). Adult sex ratio (proportion of males in the populations) was arcsine‐transformed

**Figure 3:**
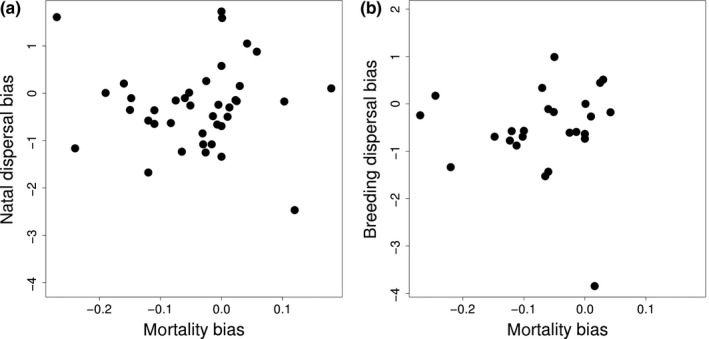
Adult mortality bias in relation to (a) natal dispersal bias (*N* = 39 species) and (b) breeding dispersal bias (*N* = 25 species). Adult mortality bias is calculated as the difference between log‐transformed male and female mortality rates

The sex bias in natal dispersal was not associated with ASR (Figures 2 and 3, PGLS, ASR: *b* = −31.740 ± 25.234; *p* = .223 ± .0001, *N* = 24 species). However, the sex bias in breeding dispersal was related to ASR: Species with male‐biased breeding dispersal exhibited female‐biased ASR (Figure 2b, PGLS, *b* = −68.376 ± 27.099; *p* = .041 ± .0001, *N* = 14 species). Although breeding dispersal bias tended to be related to mortality bias when all species were included (PGLS, *b* = 2.067 ± 1.037, *p* = .059 ± .0020, *N* = 25 species, Figure 3b), the removal of an obvious outlier species (*Calonectris diomedea*) led to remove any trend (PGLS, *b* = 1.730 ± 1.139, *p* = .262 ± .0020, *N* = 24 species).

We further investigated whether the method of ASR estimation may influence the relationship between dispersal bias and ASR. However, including ASR estimation method did not affect the results. The relationship between ASR and breeding dispersal bias remained statistically significant (PGLS, *b* = −68.144 ± 28.713, *p* = .037 ± .0001) and no effect of ASR estimation method was detectable (PGLS, *b* = −0.272 ± 0.192, *p* = .183 ± .0001, *N* = 14 species).

Finally, the exclusion of species from hunted populations did not qualitatively change our results, as shown by effect sizes (measured by adjusted *R* ^2^‐values of PGLS models) and statistical tests (Appendix S5).

## 4 DISCUSSION

Our comparative analyses, using the largest dataset of sex‐biased dispersal distances in birds ever compiled, produced two new major findings. First, while we found support for previous results that both natal and breeding dispersal are female‐biased (Greenwood, 1980; Mabry et al., 2013; Trochet et al., 2016), we provided evidence that natal and breeding dispersal biases were not related to each other. This implies that sex differences in these two types of dispersal may be driven by different biological mechanisms: the primary function of natal dispersal is to establish home ranges for all activity types and resources, whereas breeding dispersal occurs at substantially shorter time scales and focus predominantly on mating opportunities. For example, while ASR is related to breeding dispersal, we did not find evidence for a similar association with natal dispersal (see below). Although further studies are needed to explore why sex differences in these two types of dispersal behavior are decoupled from each other, an important implication is that analyses of sex‐specific dispersal should distinguish between natal and breeding dispersal. Pooling them in a single analysis may mask or bias their relationships to ecological or life‐history variables.

We also reported as a major finding that breeding dispersal—but not natal dispersal—was associated with ASR: The male‐bias in breeding dispersal distances increased with increasingly female‐biased ASR. This pattern is the opposite to the expected increase in the intensity of mating competition with the increase of the number of same‐sex competitors (see Section 1), and the reason is unclear. One explanation for this relationship may be that in species with sex‐biased ASR, individuals of the less common sex that have a high chance for finding new mates seek out mating opportunities more frequently than members of the more common sex, resulting in frequent movements by the former sex. Furthermore, earlier comparative analyses showed that in birds the rare sex tends to provide less parental care than members of the common sex (Liker, Freckleton, & Székely, 2013; Liker et al., 2015), which reduces local mating opportunity for the rare sex after members of the common sex become occupied with offspring care. Thus, it may be profitable for noncaring members of the rare sex to seek for additional mates elsewhere. The mating patterns and sex‐specific movements of some shorebirds seem to conform to this scenario. For example, ASR is male‐biased in Kentish and snowy plovers (*Charadrius alexandrinus, C. nivosus*), males typically provide parental care after hatching, and females have more breeding opportunities than males (Kosztolányi, Barta, Küpper, & Székely, 2011; Stenzel et al., 2011; Székely & Lessells, 1993). In those species, females also disperse at longer distances (up to 1,140 km) between breeding attempts and more frequently than males (Stenzel et al., 1994). Similarly, a recent study of the polygynous pectoral sandpiper (*Calidris melanotos*) that exhibits female‐only parental care showed that males move huge distances (up to 13,045 km) during the breeding season and can sample more than 20 different breeding sites when seeking mating opportunities (Kempenaers & Valcu, 2017). ASR is not known in this latter species, but females tend to outnumber displaying males in the breeding areas (Farmer, Holmes, & Pitelka, 2013), conforming to the general pattern that polygyny is usually associated with female‐biased ASR (Liker, Freckleton, & Székely, 2014; Liker et al., 2013). Thus, the missing link between ASR and natal dispersal is likely the result of that mechanisms related to searching for mating opportunities are not expected to influence natal dispersal bias.

An alternative explanation is that the negative association between ASR and sex‐biased dispersal is caused by females that disperse to avoid male harassment in populations with male‐biased ASRs. This hypothesis is supported by experimental studies, which indicate that male aggression toward females increases as a result of increased male–male competition in populations with male‐biased ASRs (Chapman, Arnqvist, Bangham, & Rowe, 2003; Le Galliard, Fitze, Ferrière, & Clobert, 2005), and this may be the main driver of female dispersal in some butterfly populations (Hovestadt & Nieminen, 2009). This process is hypothesized to be the evolutionary outcome of a sexual conflict over mating and reproduction tactics, resulting in adaptations that benefit males (in the short term) but not females (Le Galliard et al., 2005). Although sexual coercion is recognized as one of the key forces of sexual selection along with mate choice and mate competition and seems to be widespread in invertebrates, female harassment is known to exist in only a limited set of taxa in birds, for instance in waterbirds (Black, Choudhury, & Owen, 1996; McKinney, 1986) and passerines (Westcott, 1997). However, natal philopatry is known to be female‐biased in Anseriformes, likely indicating male‐biased dispersal patterns (Anderson, Rhymer, & Rohwer, 1992), especially in Anatidae, where female philopatry is typically greater than that of males (Rohwer & Anderson, 1988). These findings fail to support the hypothesis stating that female‐biased dispersal is likely to be driven by male harassment in birds.

Alternatively, biased ASR may be a consequence of sex‐biased breeding dispersal if the latter induces sex‐specific mortality, for example through energetic or predation costs of dispersal (Bonte et al., 2012; Clutton‐Brock, 2016). However, this hypothesis is not supported by our results. This finding is in contrast with the conclusion of a recent review using an independent dataset of 42 bird species, which reported that mortality is biased toward the further dispersing sex (Payevsky, 2016). However, this latter study was based on qualitative data only and did not analyze the relationships statistically.

ASR may also be associated with both natal and breeding sex‐biased dispersal if the latter is related to sex allocation, as predicted by some theoretical models, which concluded that sex‐specific dispersal is not a simple fixed process but varies in response to complex spatial and temporal patterns (Guillon & Bottein, 2011; Bonte et al. (2012). These models investigated sex allocation (i.e., the relative production of male or female offspring) and could only explain the association between the magnitude of the dispersal bias and ASR if sex allocation is associated with ASR. However, Székely et al. (2014b) did not find any detectable association between ASR and hatching sex ratio across bird species, which suggests that the relationship between sex allocation and ASR is weak.

Our findings differ from Pipoly et al.'s (2015) results of an absence of relationship between the magnitude of dispersal bias and ASR across birds. Although this latter study did not separate natal from breeding dispersal (they used dispersal data compiled by Mabry et al., 2013), its preliminary results using qualitative data on sex‐biased dispersal from a wider taxonomic range (tetrapods: amphibians, reptiles, birds, and mammals) supported that male‐biased ASR is associated with female‐biased dispersal (Supplementary Material 1 in Pipoly et al., 2015).

To investigate the exact link between dispersal bias and adult sex ratios, experimental investigations would be needed. However, less than a handful of studies are available on experimental manipulation of local sex ratios. For instance, Le Galliard et al. (2005) manipulated a population of common lizards (*Lacerta vivipara*) and showed that male mortality and emigration rates were not higher under male‐biased ASR, in contrast to the expectation. Similarly, Trochet et al. (2013) experimentally manipulated sex ratios in metapopulations of butterflies and failed to observe any sex‐biased dispersal, although sex ratio manipulations were expected to influence mate search tactics. This study concluded that female harassment by males and male–male competition might be more important mechanisms for the dispersal of both sexes than searching for a mating partner.

Sexual selection has been repeatedly assumed to be a major driver of sex‐biased dispersal (Clarke et al., 1997; Greenwood, 1980; Mabry et al., 2013; Trochet et al., 2016), although phylogenetic analyses performed so far generally failed to support this expectation (but see Trochet et al., 2016). We did not find any support for this expectation that sex‐biased dispersal is related to sexual selection, although we used several different metrics including mating system, frequency of extra‐pair paternity, relative testis size, sexual size dimorphism, and plumage dimorphism. In contrast to Trochet et al. (2016), we also failed to detect any association between the type of parental care and sex‐specific dispersal patterns. The discrepancy between studies might be related to the larger taxonomic range used by Trochet et al. (2016), which encompassed invertebrates and vertebrates other than birds, and also to the lack of separation of natal and breeding dispersal data. Additionally, Trochet et al. (2016) employed two binary variables to describe parental care in a way that was suitable for their diverse taxonomic coverage including mammals, birds, reptiles, and amphibians, whereas we used more fine‐scaled care variables that were specifically developed for birds. Therefore, we can safely conclude that there is currently no robust support for any role of sexual selection in the magnitude of sex differences in dispersal across bird species.

In conclusion, we found that dispersal distances were markedly longer in females than in males across birds and that sex‐biased breeding dispersal, but not natal dispersal, was positively associated with adult sex ratios. We call for follow‐up studies both in other taxonomic groups and in within single‐species to assess the possible causes of this relationship.

## CONFLICT OF INTEREST

None declared.

## AUTHORS' CONTRIBUTIONS

AL, TS, and ZV conceived the ideas and designed methodology; GK and ZV collected the data; ZV and AL analyzed the data; ZV, AL, and TS led the writing of the manuscript. All authors contributed critically to the drafts and gave final approval for publication.

## DATA ACCESSIBILITY

Upon acceptance, the full dataset will be published at a public server of University of Debrecen (http://www.openbiomaps.org).
