# Supplementary material for: Sex‐biased breeding dispersal is predicted by social environment in birds
Source: Ecol Evol. 2018 Jun 21;8(13):6483–91. doi: 10.1002/ece3.4095 (PMC6053579; doi:10.1002/ece3.4095)

**Appendix S1**. Sex-specific natal as well as breeding dispersal distances and literature resources used in the analyses.

This content will be available online, upon acceptance.

**Appendix S2.** References for literature resources of sex-specific natal and breeding dispersal data.

1.Arcese P., 1989. Intrasexual competition, mating system and natal dispersal in song sparrows. Animal Behaviour 38:958-979 .

2.Baker M. C., Mewaldt L. R., 1978. Song dialects as barriers to dispersal in white-crowned sparrows, *Zonotrichia leucophrys nuttalli*. Evolution 32:712-722.

3.Baker M., Nur N., Geupel G. R., 1995. Correcting biased estimates of dispersal and survival due to limited study area: theory and an application using wrentits. The Condor 97:663-674.

4.Barros Á.,Álvarez D., Velando A., 2013. Climate influences fledgling sex ratio and sex-specific dispersal in a seabird. Plos One doi:10.1371/journal.pone.0071358.

5.Bastian H-V. 1992. Breeding and natal dispersal of whinchats *Saxicola rubetra*. Ringing & Migration 13:13-19.

6.Bensch S., Hasselquist D., 1991. Territory infidelity in the polygynous great reed warbler Acrocephalus arundinaceus: the effect of variation in territory attractiveness. Journal of Animal Ecology 60:857-871.

7.Berger A. J., Radabaugh B. E., 1968. Returns of kirtland's warblers to the breeding grounds. Journal of ornithological investigation 39:161-186

8.Botsch Y., Arlettaz R., Schaub M. 2012. Breeding dispersal of eurasian hoopoes (*Upupa epops*) within and between years in relation to reproductive success, sex, and age. The Auk 129:283-295

9.Brink V. van den, Dreiss A. N., Roulin A. 2012. Melanin-based coloration predicts natal dispersal in the barn owl, *Tyto alba.* Animal Behaviour 84:805-812

10.Byholm P., Saurola P., Lindén H., Wickman M., 2003. Causes of dispersal in northern goshawks (*Accipiter gentilis*) in finland. The Auk 120:706-716.

11.Cadahía L., López-López P., Urios V., Soutollo Á.,Negro J. J., 2009. Natal dispersal and recruitment of two bonelli’s eagles *Aquila fasciata*: a four-year satellite tracking study. Acta Ornithologica 44:193-198

12.Caizergues A., Ellison L., 2002. Natal dispersal and its consequences in black grouse *Tetrao tetrix*. Ibis 144:478-487

13.Charmantier A., Buoro M., Gimenez O., Weimerskirch H., 2011. Heritability of short-scale natal dispersal in a large-scale foraging bird, the wandering albatross. Journal of Evolutionary Biology 24:1187-1496.

14.Chernetsov N., Chromik W., Dolata P. T., Profus P., Tryjanowski P., 2006. Sex-related natal dispersal of white storks (*Ciconia ciconia*) in Poland: how far and where to? The Auk 123:1103-1109.

15.Cilimburg A. B., Linberg M. S., Tewksbury J. J., Hejl S. J., 2002. Effects of dispersal on survival probability of adult yellow warblers (*Dendroica petechia*). The Auk 119:778-789.

16.Cline M. H., Strong A. M., Sillett T. S., Rodenhouse N. L., Holmes R. T.,2013. Correlates and consequences of breeding dispersal in a migratory songbird. The Auk 130:742- 752.

17.Cockburn A., Osmond H: L:, Mulder R. A., Green D. J., Double M. C., 2003. Divorce, dispersal and incest avoidance in the cooperatively breeding superb fairy-wren *Malurus cyaneus.* Journal of Animal Ecology 72:189-202.

18.Craig H. R., Kendall S., Wild T. Powell A. N. 2015. Dispersal and survival of a polygynandrous passerine. The Auk 132:916-925.

19.Dietrich V. C. J., Schmoll T., Winkel W., Lubjuhn T., 2003. Survival to first breeding is not sex-specific in the coal tit (*Parus ater*). Journal of Ornithology 144:148-156.

20.Dingemanse N. J., Both C., Noordwijk A. J. van, Rutten A. L., Drent P. J., 2003. Natal dispersal and personalities in great tits (*Parus major*) The Royal Society 741-747.

21.Drilling N. E., Thompson C. F., 1988. Natal and breeding dispersal in house wrens (*Troglodytes aedon*). The Auk 105:480-491

22.Dunn P. O., Braun C. E., 1985. Natal dispersal and lek fidelity of sage grouse. American Ornithologists' Union 102:621-627.

23.Eden S. F., 1986 Natal philopatry of the magpie *Pica pica.* Ibis 129:477-490.

24.Eikenaar C., Richardson D., S., Brouwer L., Komdeur J.,2008. Sex biased natal dispersal in a closed, saturated population of seychelles warblers, Acrocephalus sechellensis. Journal of Avian Biology 9:73-80.

25.Fajardo N., Strong A. M., Perlut N. G., Buckley N. J., 2009. Natal and breeding dispersal of bobolinks (*Dolichonyx oryzivorus*) and savannah sparrows (*Passerculus sandwichensis* ) in an agricultural landscape. The Auk 126:310-318.

26.Ferrer M. 1993. Ontogeny of dispersal distances in young spanish imperial eagles. Behavioural Ecology and Sociobiology 32:259-263.

27.Fies M. L., Puckett K. M., Larson-Brogdon B., 2002. Breeding season movements and dispersal of northern bobwhites in fragmented habitats of Virginia. in S. J. DeMaso, W.P. Kuvlesky, Jr., F. Hernandez, and M. E. Berger, eds. Quail V: Proceedings of the Fifth National Quail Symposium.

28.Fisher H. I., 1971. Experiments on homing in laysan albatrosses, *Diomedea immutabilis.* The Condor 73:389-400.

29.Forero M. G., Donázat J. A., Blas J., Hiraldo F., 1999. Causes and consequences of territory change and breeding dispersal distance in the black kite. Ecology 80:1298-1310.

30.Fraga R. M. 1991. The social system of a communal breeder, the bay-winged cowbird *Molothrus badius.* Ethology 89: 195-210.

31.Giesen K. M., Braun C. E., 1993. Natal dispersal and recruitment of juvenile white-tailed ptarmigan in Colorado. The Journal of Wildlife Management 57:72-77.

32.Gratto C. L., Morrison R. I.G., Cooke F., 1985. philopatry, site tenacity, and mate fidelity in the semipalmated sandpiper. The Auk 102:16-24.

33.Hansson B., Bensch S., Hasselquist D., Nielsen B., 2002. Restricted dispersal in a long-distance migrantbird with patchy distribution, the great reed warbler. Oecologia 130:536-542.

34.Harvey P. H., Greenwood P. J., Perrins C. M., 1979. Breeding area fidelity of great tits (*Parus major*) Journal of Animal Ecology 48:305-313.

35.Hines J. E. 1986. Survival and reproduction of dispersing blue grouse. The Condor 88:43-49.

36.Holmes R. T. 1971. density, habitat, and the mating system of the western sandpiper (*Calidris mauri*). Oecologia 7:191-208.

37.Jackson D. B. 1994. Breeding dispersal and site-fidelity in three monogamous wader species in the Western Isles, U.K. Ibis 136:463-473.

38.Jamieson I. G., Zwickel F. C., 1982. Dispersal and site fidelity in blue grouse. Canadian Journal of Zoology 61:570-573.

39.Jenkins D., Watson A., Miller G. R., 1967. Population fluctuations in the red grouse *Lagopus lagopus scoticus.* Journal of Animal Ecology 36:97-122.

40.Johns B. W., Gossen P. J., Kuyt E., Moore L. C., 2005. Philopatry and dispersal in whooping cranes. North American Crane Workshop Proceedings 9:117-125.

41.Koenig W. D., Hooge P. H., Stanback M. T., Haydock J., 2000. Natal dispersal in the cooperatively breeding acorn woodpecker. The Condor 102:492-502.

42.Korpimaki E., Lagerström M., Saurola P., 1987. Field evidence for nomadism in Tengmalm's owl, *Aegolius funereus*. Ornis Scandinavica 18:1-4.

43.Lessels C.M. 1983. Natal and breeding dispersal of canada geese *Branta canadensis*. Ibis 127:31-41.

44.López-López P., Zuberogoita Í., Gil J. A., 2013. Philopatry, natal dispersal, first settlement and age of first breeding of bearded vultures *Gypaetus barbatus* in central Pyrenees. Bird Study 60:555-560.

45.Martin C. A., Alonso J. C., Palacin C., Magana M., Martin B., 2008. Natal dispersal in great bustards: the effect of sex, local population size and spatial isolation. Journal of Animal Ecology 77:326-334.

46.Matthysen E., Schmidt K-H., 1987 Natal dispersal in the nuthatch. Ornis Scandinavica 18:313-316.

47.Mearns R., Newton I., 1984. Turnover and dispersal in a peregrine (*Falco peregrinus*) population. Ibis 126:347-355.

48.Mészáros A. L., Kajdocsi Sz., Szentirmai I., Komdeur J., Székely T., 2006. Breeding site fidelity in penduline tit Remiz pendulinus in Southern Hungary. European Journal of Wildlife Research 52:39-42.

49.Miller K. E., SmallwoodJ. A., 1997. Natal dispersal and philopatry of southeastern american kestrels in Florida. The Wilson Bulletin 109:226-232.

50.Moore W. S., Dolbeer A. R., 1989. The use of banding recovery data to estimate dispersal rates and gene flow in avianspecies: case studies in the red-winged blackbird and common grackle. American Ornithologycal Society 91:242-253.

51.Morton M. L., 1992. Effects of sex and birth date on premigration biology, migration schedules, return rates and natal dispersal in the mountain white-crowned sparrow. The Condor 94:117-133

52.Nagata H., 1993. The structure of a local population and dispersal pattern in the Styan's grasshopper warbler, *Locustella pleskei*. Ecologycal Research 8:1-9.

53.Negro J. J., Hiraldo F., Donazar J. A., 1997. Causes of natal dispersal in the lesser kestrel: Inbreeding avoidance or resource competition? Journal of Animal Ecology 66:640-648.

54.Nesbitt S. A., Schwikert T., Folk M. J., 2002. Natal dispersal in florida sandhill cranes. The Journal of Wildlife Management 2:349-352.

55.Newton I., Davis P. E., Davis J. E., 1987. Age of first breeding, dispersal and survival of red kites *Milvus rnilvus* in Wales. Ibis 131:16-21.

56.Nilsson J-A. 1989. Causes and consequences of natal dispersal in the marsh tit, *Parus palustris*. Journal of Animal Ecology 58:619-636.

57.Okill J. D., 1992 Natal dispersal and breeding site fidelity of red‐throated divers *Gavia stellata* in Shetland. Ringing & Migration 13:57-58.

58.Osorio-Beristain M., Drummond H., 1993. Natal dispersal and deferred breeding in the blue-footed booby. The Auk 110:234-239.

59.Part T. 1990. Natal dispersal in the collared flycatcher: Possible causes and reproductive consequences. Ornis Scandinavica 21:83-88.

60.Pasinelli G., Müller M., Schaub M., Jenni L., 2007. Possible causes and consequences of philopatry and breeding dispersal in red-backedshrikes *Lanius collurio*. Behavioral Ecology and Sociobiology 61:1061-1074

61.Payne R. B., 1991. Natal dispersal and population structure in a migratory songbird, the indigo bunting. Evolution 45:49-62.

62.Picozzi N. 1984. Breeding biology of polygynous hen harriers *Circus c. cyaneus* in Orkney. Ornis Scandinavica 15:1-10.

63.Pipoly I., Bókony V., Kirkpatrick M., Donald P. F. Székely T., 2005. The genetic sex-determination system predicts adult sex ratios in tetrapods. Nature 527:91-94.

64.Plissner J. H., Gowaty P. A. 1996. Patterns of natal dispersal, turnover and dispersal costs in eastern bluebirds. Animal Behavior 51:1307-1322.

65.Potti J., Montalvo S., 1991. Return rate, age at first breeding and natal dispersal of pied flycatchers F*icedula hypoleuca* in Central Spain. Ardea 79.

66.Pyle P. 2001. Age at first breeding and natal dispersal in a declining population of cassin's auklet. The Auk 118:996-1007.

67.Richardson K., Ewen J. G., Armstrong D. P., Hauber M. E., 2010. Sex-specific shifts in natal dispersal dynamics in a reintroduced hihi population. Behaviour 147:1517-1532.

68.Robinson J. A., Oring L. W., 1997. Natal and breeding dispersal in american avocets. The Auk 114:416-430

69.Rosenfield N., Bielefeldt J., 1992. Natal dispersal and inbreeding in the cooper's hawk. The Vision Bulletin 104:182-184.

70.Rosier J. F., Ronan N. A., Rosenberg D.K. 2006. Post-breeding dispersal of burrowing owls in an extensive california grassland. The American Midland Naturalist 155:162-167.

71.Russel E. M., Rowley I., 1993. Philopary and dispersal: competiton for terrytory vacansies in the splendid fairy-wren, *Malurus splendens*. Animal Behaviour 45:519-539.

72.Sankamethawee W., Hardesty B. D., Gale G. A., 2010. Sex-bias and timing of natal dispersal in cooperatively breeding puff-throated bulbuls *Alophoixus pallidus.* Journal of Ornithology 151:779-789.

73.Schiegg K., Daniels S. J., Walters J. R., Priddy J. A., 2006. Inbreeding in red-cockaded woodpeckers: Effects of natal dispersal distance and territory location. Biological Conservation 131:544-552.

74.Sedgwick J. A. 2004. Site fidelity, territory fidelity, and natal philopatry in willow flycatchers (*Empidonax traillii*). The Auk 121:1103.

75.Shields W. M., 1984. Factors affecting nest and site fidelity in adirondack barn swallows (*Hirundo rustica*). The Auk 4:780-789.

76.Skrade D. B., Dinsmore S.J., 2010. Sex-related dispersal in the mountain plover (*Charadrius montanus*) The Auk 127:671-677.

77.Small R. J., Rusch D. H. 1989. The natal dispersal of ruffed grouse. The Auk 106:72-79.

78.Shutler D., Clark R. G., 2003. Causes and consequences of tree swallow (*Tachycineta bicolor*) dispersal in saskatchewan. The Auk 120:619-631.

79.Spear L. B., Pyle P. Nur N., 1998. Natal dispersal in the western gull proximal factors and consequences. Ecology 67:165-179.

80.Stenzel L. E., Page G. W., Warrine J. C., Warriner J. S. George D. E., Eyster C. R., Ramer B. A., Neuman K. K., Sandercock B. K. 2007. Survival and natal dispersal of juvenile snowy plovers (*Charadrius alexandrinus*) in Central Coastal California. The Auk 124:1023-1036.

81.Strickland D., 1990 Juvenile dispersal in gray jays: dominant brood member expels siblings from natal territory. Canadian Journal of Zoology 69:2935-2945.

82.Thibault J-C 1993. Natal philopatry in the cory's shearwater (*Calonectris d. diomedea*) on Lavezzi island, Corsica. Colonial Waterbirds 16:77-82.

83.Tyle S. J., Ormedor S.J., Lewis J. M. S., 1990. The post-natal and breeding dispersal of welsh dippers *Cinclus cinclus*. Bird Study, 37:18-22.

84.Wheel Wright N. T., Mauck R. A., 1998. Philopatry, natal dispersal, and inbreeding avoidance in an island population of savannah sparrows. Ecology 79:755-767.

85.Wilcox L., 1959. A twenty year banding study of the piping plover. The Auk 76.

86.Wiklund C. G., 1996. Determinants of dispersal in breeding merlins (*Falco Columbarius*). Ecology 77:1920-1927.

87.Winkler D. W., Wrege P. H., Allen P. E., Kast T. L., Senesac P., Wasson M., F., Sullivaan P. J., 2005. The natal dispersal of tree swallows in a continuous mainland environment. Journal of Animal Ecology 74:1080-1090.

88.Whitfield D. P., Douse A., Evans R. J., Grant J., Love J., Mcleod D. R. A., Reid R., Wilson J. D., 2009. Natal and breeding dispersal in a reintroduced population of white‐tailed eagles *Haliaeetus albicilla*. Bird Study 56:177-186.

89.Wlodarczyk R., Wieloch M., Czyz S., Dolata P., T., Minas P., 2013. Natal and breeding dispersal in mute Swans *Cygnus olor*: influence of sex, mate switching and reproductive success. Acta Ornithologica. 48:237–244

**Appendix S3**. PRISMA-diagram of methodological approach for analysing sex-biased dispersal patterns in birds.


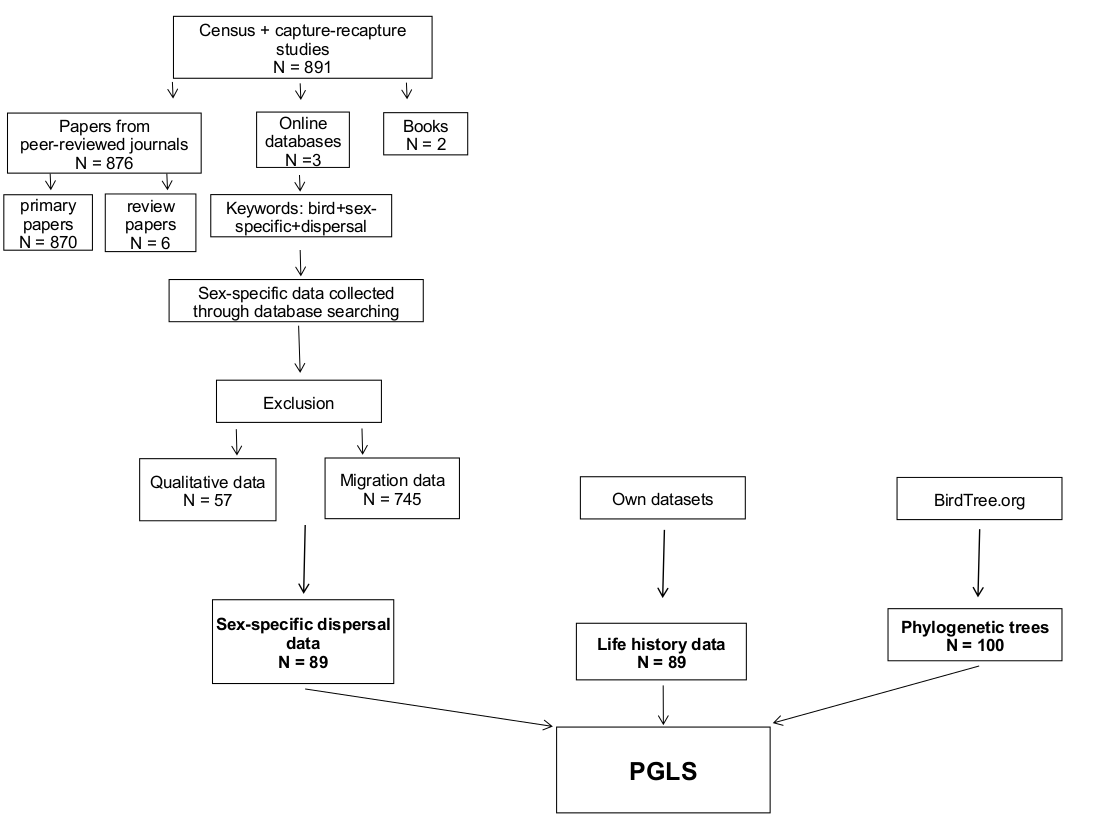


**Appendix S4.** Phylogenetic signal in the sign of sex-biased dispersal, measured by λ-values retrieved from PGLS-models and D-statistics, where D stands for the estimated D-statistic and P1 yields the result of testing whether D is different from 1.0, indicating phylogenetic randomness. All of these metrics are calculated across the 100 phylogenetic trees applied in the PGLS-models.

|  | **Natal dispersal bias** | | **Breeding dispersal bias** | |
| --- | --- | --- | --- | --- |
| **Predictors** | **λ** | **D-statistic**  D  P1 | **λ** | **D-statistic**  D  P1 |
| **Sexual selection** |  |  |  |  |
| Social mating system | <0.001 | 0.828  0.245 | <0.001 | 1.473  0.814 |
| Testis size | 0.089 | 0.625  0.147 | <0.001 | 1.221  0.615 |
| Extra-pair broods | <0.001 | 1.195  0.657 | <0.001 | 1.399  0.636 |
| Parental care | <0.001 | 0.897  0.329 | <0.001 | 1.287  0.709 |
| Sexual size dimorphism | 0.060 | 0.695  0.143 | <0.001 | 1.069  0.532 |
| Sexual dichromatism | <0.001 | 1.051  0.519 | 0.027 | -0.474  0.059 |
| **Social environment** |  |  |  |  |
| Adult sex ratio | 0.400 | 0.457  0.118 | <0.001 | 1.594  0.702 |
| Mortality bias | Not tested | Not tested | <0.001 | 1.423  0.738 |

**Appendix S5**. Performance of PGLS-models fitted on datasets including and excluding species from hunted populations.

|  | **Natal dispersal bias** | | **Breeding dispersal bias** | |
| --- | --- | --- | --- | --- |
| **Predictors** | **Hunted species included**  b, R^2^  p, Number of species | **Hunted species excluded**  b, R^2^  p, Number of species | **Hunted species included**  b, R^2^  p, Number of species | **Hunted species excluded**  b, R^2^  p, Number of species |
| **Sexual selection** |  |  |  |  |
| Social mating system | 0.026, -0.015  0.476, 58 | 0.068, -0.06  0.424, 51 | -0.013, -0.028  0.764, 28 | -0.129, 0.013  0.334, 25 |
| Testis size | -0.076, -0.024  0.694, 36 | -0.104, -0.028  0.625, 29 | -0.297, 0.051  0.098, 29 | -0.420, 0.046  0.081, 23 |
| Extra-pair broods | -0.443, -0.004  0.361, 36 | -0.643, 0.029  0.182, 30 | 0.387, -0.043  0.095, 27 | 0.244, -0.047  0.936, 24 |
| Parental care | 0.041, -0.016  0.798, 56 | 0.022, -0.005  0.387, 49 | -0.151, -0.007  0.256, 30 | -0.080, -0.017  0.477, 26 |
| Sexual size dimorphism | 0.401, -0.016  0.693, 55 | 0.516, -0.017  0.635, 48 | 0.234, -0.021  0.551, 38 | 0.263, -0.002  0.339, 31 |
| Sexual dichromatism | 0.033, -0.038  0.710, 25 | -0.006, -0.059  0.966, 19 | 0.045, -0.058  0.559, 22 | 0.312, 0.026  0.332, 16 |
| **Social environment** |  |  |  |  |
| Adult sex ratio | -31.740, 0.025  0.223, 24 | -28.06, -0.002  0.343, 18 | -68.376, 0.335  0.041, 14 | -80.478, 0.297  0.031, 13 |
| Mortality bias | Not tested | Not tested | 2.067, 0.037  0.059, 25 | 1.576, -0.002  0.338, 22 |

**Appendix S6.** Relationship between natal versus breeding dispersal bias.


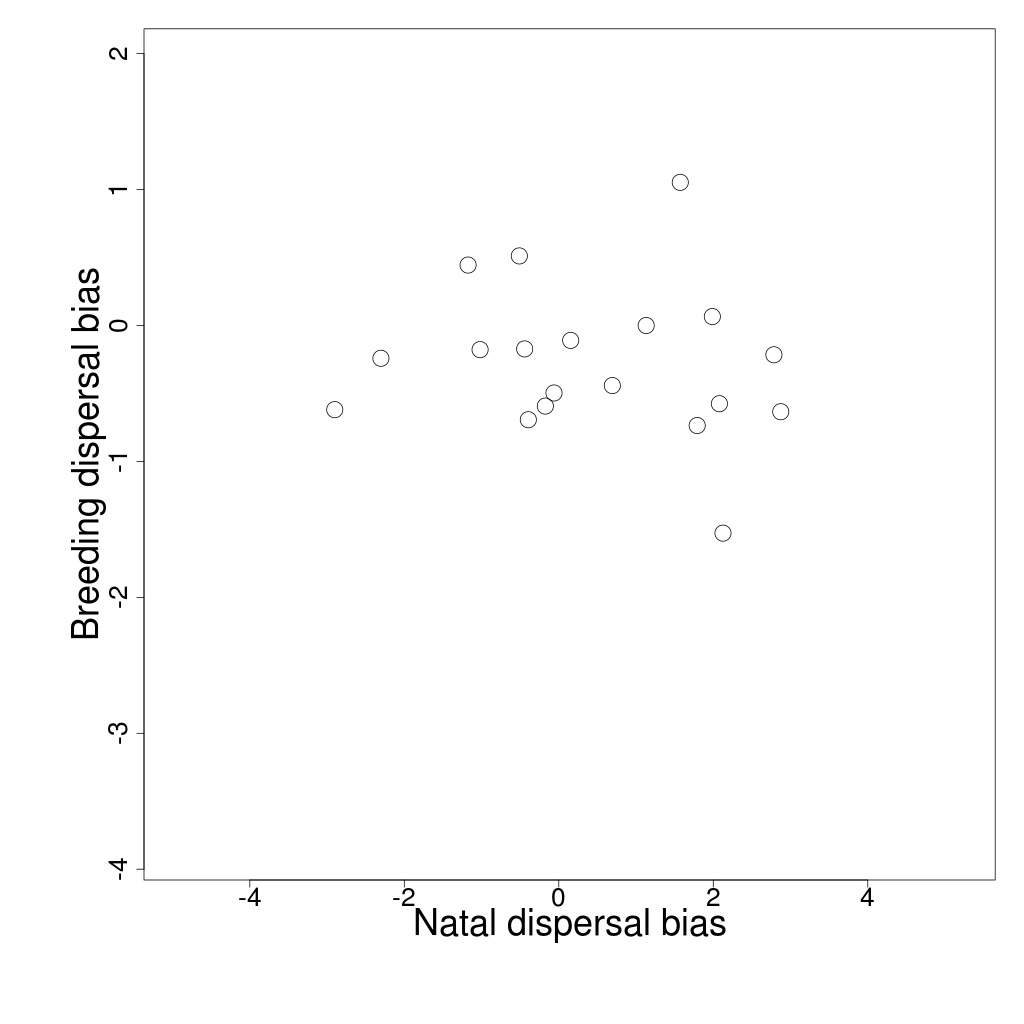

Supplement: Supplementary file 1 [file ECE3-8-6483-s001.doc]
